# A clinical follow‐up of omalizumab in routine treatment of allergic asthma monitored by CD‐sens

**DOI:** 10.1002/iid3.225

**Published:** 2018-05-07

**Authors:** S. Gunnar O. Johansson, Gunnar Lilja, Jenny Hallberg, Anna Nopp

**Affiliations:** ^1^ Department of Clinical Science and Education Södersjukhuset and Karolinska Institutet Stockholm Sweden; ^2^ Sachś Children and Youth Hospital Södersjukhuset Stockholm Sweden; ^3^ Institute of Environmental Medicine Karolinska Institutet Stockholm Sweden

**Keywords:** Basophils, IgE‐antibody, omalizumab

## Abstract

**Introduction:**

Omalizumab has been available for treatment of allergic asthma for more than a decade and thus, its efficacy in routine treatment was of interest to evaluate. Basophil allergen threshold sensitivity (CD‐sens) has been shown to correlate with the bronchial allergen threshold sensitivity and can be used to objectively measure omalizumab treatment efficacy. We aimed to evaluate the effect of omalizumab treatment of allergic asthma by CD‐sens, as an objective marker of the IgE‐mediated inflammation, and related to SPT, spirometry, FeNO, Asthma Control Questionnaire (ACQ), and Global Evaluation of Treatment Effectiveness (GETE).

**Methods:**

Thirty‐two patients were treated with omalizumab for 16 weeks. CD‐sens was used to define the response and related to clinical parameters. If CD‐sens was negative (<0.1) (CD‐sens low Group) the patient continued with the standard dose. If CD‐sens was ≥0.1 (CD‐sens high Group) a second 16 weeks period with 25–50% dosage increase was started and evaluated after a total of 32 weeks.

**Results:**

Nine of 32 patients became CD‐sens negative after treatment (CD‐sens start: 8.0; 16 weeks: <0.01) and regarded as successful. 15/23 were unsuccessful (CD‐sens start: 13; 16 weeks: 1.65) and the omalizumab dose was increased. CD‐sens decreased significantly (*p* < 0.05) and further 3/15 patients became CD‐sens negative (CD‐sens at 32 weeks: 0.5). There was a significantly smaller IgE‐ab fraction (IgE‐ab/IgE) in the CD‐sens low versus the CD‐sens high Group (*p* < 0.0001). A significant decrease in ACQ was seen in both groups after 16 weeks treatment (*p* = 0.05 and 0.01, respectively). No significant changes could be detected for the other clinical parameters.

**Conclusion:**

By the use of the objective laboratory method CD‐sens, which effectively measure the direct effect of omalizumab, that is, the IgE‐mediated part of the allergic asthma, in combination with clinical parameters it might be possible to more effectively monitor and treat IgE‐mediated allergic asthma.

## Introduction

Allergic diseases such as allergic asthma and rhinitis are caused by an immune inflammation dependent on the allergenic material in pollen, dander and food, that the patient is exposed to, and IgE antibodies (IgE‐ab) produced by repeated stimulation of the allergen.

Some 15 years ago a new approach to the treatment of allergic asthma was introduced 1 . A humanized mouse monoclonal antibody to human IgE is injected and blocks the structure on the Fc‐part responsible for binding to the FcϵI‐receptor on the surface of mast cells and basophilic granulocytes. As a consequence an IgE‐induced inflammation cannot be initiated. The monoclonal anti‐IgE named omalizumab was registered for treatment of allergic asthma and became commercially available (Xolair, Novartis, Switzerland).

Omalizumab is used in the clinical routine to treat severe cases of allergic asthma and chronic urticaria 2 . The registered omalizumab dosage is based on the patient serum IgE concentration and the patient body weight and should press down serum IgE to approximately 10 kU/L. However, if the recommended dosage is given as many as 39% of the patients have been reported to not respond satisfactory 3 and the evaluation of lung function parameters over time in omalizumab responder and non‐responder patients remains inconclusive 4 . Thus, several studies indicate that lung function parameters are not sensitive enough to mirror the treatment effectiveness of omalizumab in patients with asthma 5, 6, 7, 8 .

Basophil allergen threshold sensitivity, CD‐sens, where a dose‐response allergen stimulation of the basophils is performed, has previously been shown to accurately measure the IgE‐mediated component of the allergic asthma and is not influenced by the hyper‐reactivity which is driven by other factors 9 . The primary outcome for omalizumab, to block the initiation of the IgE‐mediated allergic inflammation, is effectively measured and monitored by CD‐sens. Thus, CD‐sens is a simple and side‐effect free alternative to bronchial allergen threshold challenge.

The objective of this “real life” study was to evaluate the effect of omalizumab treatment of allergic asthma by CD‐sens, as an objective marker of responders/non‐responders regarding the IgE‐mediated inflammation, and to relate the outcome to SPT and clinical parameters like FEV1, FEV1/FVC, Fractional exhaled Nitric Oxide (FeNO), Asthma Control Questionnaire (ACQ), and Global Evaluation of Treatment Effectiveness (GETE).

## Methods

### Study population

Physicians treating patients with allergic asthma and intending to introduce omalizumab were invited to participate in a treatment follow‐up study evaluating omalizumab efficacy. Eighteen physicians and thirty‐two patients were included (Table 1 ). All patients had an inadequate asthma control despite treatment with ciclesonide (*n* = 4), high doses of budesonide/fluticasone combined with long‐acting beta‐2‐agonists (*n* = 28), and/or anti‐leukotriens (*n* = 11). All patients were multi‐sensitized verified with a positive SPT and detectable levels of IgE‐ab. For each patient the most important allergen triggering the asthma was selected and followed by CD‐sens and SPT.

**Table 1:** Patient characteristics

Patient ID	Age years	Male (M)/Female (F)	Allergen	IgE (kU/L)	IgE‐ab (kU_A_/L)	IgE‐ab (%)
CD‐sens low group						
1	12	F	Dpt f	522	>100	>20
5	14	M	Dog	776	47	5.9
10	52	F	Cat	150	0.5	0.3
12	26	F	Birch	380	12	3.2
19	58	M	Cat	140	1.4	1.0
22	49	M	Dog	120	3.9	3.3
30	30	M	Dog	820	24	2.9
31	59	F	Horse	3700	46	1.2
36	14	M	Birch	1878	32	1.7
CD‐sens high group						
2	23	F	Birch	210	60	29
3	10	M	Birch	210	250	21
4	17	M	Birch	160	36	23
6	14	F	Cat	3001	>100	>3.0
8	30	F	Birch	160	16	10
9	19	F	Dog	120	29	24
14	21	M	Dog	6341	>100	>2.0
15	12	F	Cat	280	14	5.0
16	17	F	Birch	640	87	14
17	50	M	Alternaria	180	6.1	3.4
18	46	F	Birch	320	42	13
20	25	M	Dog	70	15	21
21	11	M	Cat	1000	>100	>10
23	26	F	Birch	170	69	41
24	20	F	Birch	370	44	12
25	30	F	Birch	630	45	7.1
26	18	F	Cat	730	95	13
29	15	M	Cat	200	39	20
32	18	M	Cat	397	69	17
33	12	M	Birch	98	29	30
34	16	F	Mite	13	5.7	44
37	7	M	Birch	3700	55	1.5
38	45	M	Birch	30	0.97	3.2

The clinical follow‐up was approved by the ethics committee in Stockholm, 2014/674‐31/4, and all patients and caregivers gave their written informed consent to participate in the study.

### Study design

IgE (“total‐IgE”) and IgE‐antibodies, IgE‐ab (“specific IgE”), was analyzed before treatment and data including medical history, physical examination as well as FEV1, FEV1/FVC, exhaled NO‐measurement, and CD‐sens were analyzed before and after a 16 weeks omalizumab‐treatment period. In some patients SPT wheal size data were available. Patients or parents were asked to fill in an Asthma Control Questionnaire (ACQ) on symptoms 10 , before and after 16 weeks and a GETE 11 was performed by the physician after treatment. CD‐sens was used to evaluate the response to omalizumab. A CD‐sens of <0.1 (CD‐sens low Group) was regarded as a negative test and the treatment judged as successful, while a CD‐sens of ≥0.1 (CD‐sens high Group) was judged as unsuccessful. Omalizumab was given according to the dosing scheme registered for allergic asthma i.e. dose based on serum IgE concentration and body weight.

If CD‐sens was <0.1 (CD‐sens low Group) the patient continued with the same dose of omalizumab and no further follow up was performed. If CD‐sens was ≥0.1 (CD‐sens high Group) a second 16 weeks period with a 25–50% increase in omalizumab dosage was implemented and the clinical and immunological evaluation was repeated after these additional 16 weeks, that is, a total of 32 weeks treatment.

### Skin Prick Test

Skin Prick Test (SPT) was performed with relevant allergen extracts (Soluprick, ALK, Copenhagen, Denmark) before the start of treatment with omalizumab and after 16 and 32 weeks treatment. Histamine hydrochloride 10 mg/ml (ALK) was used as positive control and the diluent as negative control. The test was considered as positive if the wheal was ≥ 3 mm in diameter after 15 min and the degree of reactivity was defined after measuring the wheal area (mm^2^).

### Serum IgE analyses

IgE and IgE‐ab to airborne allergens (timothy, birch, cat, dog, horse, Altenaria, *Dermatophagoides farinae [Dpt f]*) were analyzed before start of treatment using ImmunoCAP® (Thermo Fisher Scientific, Uppsala, Sweden), according to the instructions of the manufacturer. The cut‐off for a positive test was set to >0.1 kU_A_/L.

### Fractional exhaled Nitric Oxide

FeNO was determined prior to spirometry, using the NIOX MINO Airway Inflammation Monitor (Aerocrine AB, Solna, Sweden) according to ATS/ERS criteria 12 . The determination of FeNO was performed before the start of treatment with omalizumab and after 16 and 32 weeks treatment. FeNO values are expressed as ppb.

### Spirometry

Spirometry was performed according to ATS/ERS criteria 13 at the same occasions as the FeNO evaluations. The highest values of Forced Vital Capacity (FVC) and Forced Expiratory Volume in 1 sec (FEV_1_) were used for analysis. The spirometry system was calibrated each day using a 3 L precision syringe.

### Basophil activation

Blood drawn into heparin tubes were sent to the diagnostic laboratory at Karolinska University Hospital in Solna, Stockholm or Uppsala University Hospital in Uppsala and analyzed within 24 h. Basophil activation was performed as previously published 14 . Basophils were stimulated with decreasing concentrations of an allergen extract (Aquagen or Soluprick, ALK) until no response was obtained (concentration range: 0.1–10,000 Standard Quality Unit (SQU)/ml for Aquagen and 0.1–10,000 Arbitrary Units (AU)/ml for Soluprick). Since the allergen extracts are not standardized for potency or allergen composition the dose response cannot be compared between different allergens but for each allergen between samples and patients. Anti‐FcϵRI (Bühlmann Laboratories AG, Schönenbuch, Switzerland) was used as positive control and RPMI (cell culture media developed at Roswell Park Memorial Institute) as negative control. To identify the basophils, cells were stained for CD203c and to detect activated basophils, the cells were stained for CD63 (Immunotech, Marseille, France) followed by analysis in a Navios flow cytometer (Beckman Coulter, Inc., Fullerton, CA). Cut‐off determining a positive test was set to 5% of CD63‐positive basophils. Patients, whose basophils after stimulation with the positive control (anti‐FcϵRI) responded with less than 5% CD63 up‐regulation, were regarded as non‐responders. Individuals with a response between 5% and 16% were classified as low responders. The cut‐off of 16% was calculated (mean 76%–3 SD) from the positive controls of an in‐house reference material of 264 allergic children and adults 15 .

### Definition of CD‐sens

To determine the basophil allergen threshold sensitivity, CD‐sens, the eliciting allergen concentration giving 50% (EC50) of maximum CD63% up‐regulation of the dose‐response curve was calculated. CD‐sens definition: the inverted value for EC50 multiplied by 100 16 . A CD‐sens of <0.1 was regarded as a negative test. CD‐sens cannot be used to evaluate allergen sensitivity among non‐ and low responders. CD‐sens was analyzed before the start of treatment with omalizumab and after 16 and 32 weeks treatment.

### Statistics

Continuous variables are presented as median and inter quartile range if not either wise stated. Spirometry values were converted to z‐scores according to Quanjer et al. 17 . Differences between pre‐ and post‐treatment samples were tested with Wilcoxon matched‐pairs signed rank test. Statistical significance was considered at a *p*‐value of <0.05. No adjustment for multiple testing has been performed. Thus, significant results should be regarded as descriptive and explorative. Statistical analyses were carried out using GraphPad Prism (Version 7.04).

## Results

### Study population

Thirty‐two asthmatic patients were treated with omalizumab for 16 weeks. Fourteen of 32 patients (44 %) were male and 15/32 (47 %) were 18 years or younger (Table 1 ). Treatment was monitored by CD‐sens, SPT (Table 2 ), FeNO, patient questionnaires (ACQ), GETE (Table 3 ), and spirometry (Table 4 ).

**Table 2:** Evaluation of CD‐sens and SPT after omalizumab treatment

		Allergen source	Allergen concentrations	Unit	CD‐sens			SPT		
Patient ID	Allergen	CD‐sens	CD‐sens	CD‐sens	Start	16 w	32 w	Start	16 w	32 w
CD‐sens low group										
1	Dpt f	Aquagen, ALK	0.1–10,000	SQU/ml	27	0.01	nt	15.9	3.1	nt
5	Dog	Aquagen, ALK	0.1–10,000	SQU/ml	5.3	0.01	nt	50.2	nt	nt
10	Cat	Aquagen, ALK	0.1–10,000	SQU/ml	6.1	0.01	nt	nt	12.5	nt
12	Birch	Aquagen, ALK	0.1–10,000	SQU/ml	161	0.01	nt	19.6	12.6	nt
19	Cat	Aquagen, ALK	0.1–10,000	SQU/ml	0.3	0.01	nt	28.3	12.6	nt
22	Dog	Aquagen, ALK	0.1–10,000	SQU/ml	8.5	0.01	nt	15.9	44.2	nt
30	Dog	Aquagen, ALK	0.1–10,000	SQU/ml	8.0	0.01	nt	63.6	19.6	nt
31	Horse	Aquagen, ALK	0.1–10,000	SQU/ml	150	0.01	nt	86.5	nt	nt
36	Birch	Aquagen, ALK	0.1–10,000	SQU/ml	3.8	0.01	nt	7.1	7.1	nt
CD‐sens high group										
2	Birch	Aquagen, ALK	0.1–10,000	SQU/ml	1786	16.6	nt	12.6	19.6	nt
3	Birch	Aquagen, ALK	0.1–10,000	SQU/ml	0.8	0.3	nt	nt	nt	nt
4	Birch	Aquagen, ALK	0.1–10,000	SQU/ml	7.9	1.1	0.5	78.5	23.7	nt
6	Cat	Aquagen, ALK	0.1–10,000	SQU/ml	78.8	2.7	0.5	44.2	12.6	15.9
8	Birch	Aquagen, ALK	0.1–10,000	SQU/ml	244	8.4	3.0	nt	nt	7.1
9	Dog	Aquagen, ALK	0.1–10,000	SQU/ml	7.2	1.7	0.2	19.6	56.7	9.6
14	Dog	Aquagen, ALK	0.1–10,000	SQU/ml	1.7	1.3	0.1	nt	nt	44.2
15	Cat	Aquagen, ALK	0.1–10,000	SQU/ml	8.8	3.4	0.01	33.2	28.3	70.9
16	Birch	Aquagen, ALK	0.1–10,000	SQU/ml	13	0.3	0.01	19.6	19.6	3.1
17	Alternaria	Soluprick, ALK	0.1–10,000	AU/ml	130	23	6.0	38.5	3.1	56.7
18	Birch	Aquagen, ALK	0.1–10,000	SQU/ml	7.7	0.3	0.6	12.6	9.6	nt
20	Dog	Aquagen, ALK	0.1–10,000	SQU/ml	9.2	0.4	nt	44.2	nt	nt
21	Cat	Aquagen, ALK	0.1–10,000	SQU/ml	2.0	0.1	0.1	33.2	9.6	9.6
23	Birch	Aquagen, ALK	0.1–10,000	SQU/ml	110	1.6	0.9	28.3	19.6	15.9
24	Birch	Aquagen, ALK	0.1–10,000	SQU/ml	2.9	0.8	nt	23.8	7.1	nt
25	Birch	Aquagen, ALK	0.1–10,000	SQU/ml	17	0.1	0.01	12.6	7.1	15.9
26	Cat	Aquagen, ALK	0.1–10,000	SQU/ml	427	0.5	0.8	19.6	12.6	9.6
29	Cat	Aquagen, ALK	0.1–10,000	SQU/ml	80	30	1.4	23.7	63.6	33.2
32	Cat	Aquagen, ALK	0.1–10,000	SQU/ml	115	4.1	0.2	269	63.6	56.7
33	Birch	Aquagen, ALK	0.1–10,000	SQU/ml	448	0.6	nt	23.7	33.2	nt
34	Mite	Aquagen, ALK	0.1–10,000	SQU/ml	3.6	0.1	nt	12.6	19.6	nt
37	Birch	Aquagen, ALK	0.1–10,000	SQU/ml	70	0.8	nt	nt	nt	nt
38	Birch	Aquagen, ALK	0.1–10,000	SQU/ml	2.6	0.5	nt	21.2	15.9	nt

SPT, skin prick test, wheal in mm^2^; w, week; SQU/ml: standard quality unit/ml; AU/ml, arbitrary unit/ml; nt, not tested.

**Table 3:** Evaluation of FeNO, ACQ, and GETE after omalizumab treatment

	FeNO			ACQ			GETE	
Patient ID	Start	16 w	32 w	Start	16 w	32 w	16 w	32 w
CD‐sens low group								
1	54	58	nt	3.2	2.8	nt	2	nt
5	14	8	nt	3.0	2.7	nt	4	nt
10	59	25	nt	4.3	3.0	nt	2	nt
12	19	5	nt	2.0	2.3	nt	2	nt
19	39	57	nt	2.5	2.1	nt	3	nt
22	19	9	nt	3.5	2.2	nt	2	nt
30	11	15	nt	3.8	1.3	nt	3	nt
31	30	21	nt	4.2	2.5	nt	3	nt
36	nt	nt	nt	5.7	4.2	nt	nt	nt
CD‐sens high group								
2	113	19	nt	2.8	0.3	nt	1	nt
3	>60	n.t	nt	4.5	1.0	nt	1	nt
4	50	18	17	1.2	0.3	nt	2	nt
6	30	16	71	2.8	0.3	0.5	nt	nt
8	11	14	Nt	0.3	0.3	0.3	3	3
9	12	8	15	1.8	1.2	0.8	3	1
14	60	9	11	1.2	0.2	0.3	2	2
15	59	34	22	5.2	2.0	1.2	2	2
16	18	7	6	1.0	0.8	0.3	3	1
17	27	11	21	4.3	2.5	1.7	2	2
18	8	9	9	2.5	0.8	0.5	2	2
20	26	15	nt	3.8	3.7	nt	3	nt
21	49	11	20	2.0	0.7	0.5	2	1
23	13	34	15	0.01	0.7	0.7	2	2
24	32	32	nt	3.0	2.5	nt	2	nt
25	24	20	26	2.2	1.8	0.5	2	2
26	13	8	8	1.0	0.2	0.1	2	2
29	11	12	16	1.7	0.8	0.5	2	2
32	5	6	7	1.2	0.5	0.3	nt	nt
33	nt	nt	nt	nt	1.2	nt	nt	nt
34	nt	nt	nt	2.8	3.8	nt	nt	nt
37	39	17	nt	4.0	2.7	nt	3	nt
38	32	27	nt	1.5	2.2	nt	2	nt

ACQ, asthma control questioner; FeNO, fractional exhaled nitric oxide; GETE, global evaluation of treatment effectiveness; nt, not tested.

**Table 4:** Evaluation of spirometry after omalizumab treatment

	FEV_1_			FVC			FEV1/FVC			Z‐score FEV_1_			Z‐score FVC			Z‐score FEV1/FVC		
Patient ID	Start	16 w	32 w	Start	16 w	32 w	Start	16 w	32 w	Start	16 w	32 w	Start	16 w	32 w	Start	16 w	32 w
CD‐sens low group																		
1	1.67	2.05	nt	1.93	2.42	nt	0.87	0.85	nt	−3.10	−2.21	nt	−3.21	−2.05	nt	−0.30	−0.62	nt
5	2.31	2.84	nt	3.36	3.50	nt	0.76	0.81	nt	−1.42	−1.09	nt	−0.64	−0.75	nt	−1.44	−0.75	nt
10	2.45	2.82	nt	nt	nt	nt	nt	nt	nt	nt	nt	nt	nt	nt	nt	nt	nt	nt
12	3.07	4.03	nt	4.13	4.24	nt	0.90	0.91	nt	0.77	1.01	nt	0.27	0.37	nt	0.72	0.93	nt
19	2.93	2.14	nt	3.79	2.95	nt	0.77	0.73	nt	−0.63	−2.35	nt	−0.51	−2.05	nt	−0.23	−0.89	nt
22	2.07	2.39	nt	2.94	3.13	nt	0.70	0.76	nt	−2.91	−2.23	nt	−2.37	−2.01	nt	−1.49	−0.60	nt
30	3.72	4.09	nt	6.24	6.47	nt	0.60	0.63	nt	−1.50	−0.83	nt	1.06	1.42	nt	−3.07	−2.69	nt
31	1.75	1.90	nt	2.19	2.48	nt	0.80	0.77	nt	−1.12	−0.60	nt	−1.11	−0.33	nt	−0.04	−0.51	nt
36	2.14	3.4	nt	1.93	2.42	nt	0.87	0.85	nt	nt	nt	nt	nt	nt	nt	nt	nt	nt
CD‐sens high group																		
2	3.46	3.69	nt	4.45	4.42	nt	0.78	0.83	nt	−3.10	−2.21	nt	0.55	0.49	nt	−1.32	−0.50	nt
3	1.70	1.97	nt	2.32	2.37	nt	0.73	0.83	nt	−1.42	−1.09	nt	1.37	1.46	nt	−2.03	−0.67	nt
4	2.33	3.12	nt	4.02	4.35	nt	0.58	0.72	nt	0.77	1.01	nt	−0.71	−0.07	nt	−3.33	−2.27	nt
6	1.62	1.90	1.78	1.88	2.00	2.01	0.92	0.88	0.85	−0.63	−2.35	−3.04	−2.74	−2.61	−2.86	0.43	−0.40	−0.80
8	nt	2.90	2.69	nt	3.79	nt	nt	0.76	nt	−2.91	−2.23	nt	nt	0.90	nt	nt	−1.31	nt
9	3.65	3.47	3.44	4.11	3.91	4.03	0.89	0.89	0.85	−1.50	−0.83	0.13	0.50	0.07	0.31	0.07	0.08	−0.44
14	3.91	3.75	4.58	5.45	nt	6.02	0.72	nt	0.76	−1.12	−0.60	−0.90	−0.88	nt	−0.10	−1.83	nt	−1.25
15	1.82	2.00	nt	2.59	2.74	Nt	0.70	0.73	nt	−0.30	0.26	nt	0.53	0.94	Nt	−2.52	−2.25	nt
16	3.82	3.96	3.84	4.04	4.07	4.36	0.95	0.93	0.88	−0.21	1.05	0.04	−0.41	−0.38	0.03	1.06	0.68	−0.12
17	2.51	3.83	3.62	4.48	4.46	4.75	0.78	0.83	0.77	−3.29	−1.60	−0.65	−0.91	−0.93	−0.46	−0.13	0.66	−0.38
18	2.95	2.66	2.81	nt	3.44	3.78	nt	0.77	0.74	−2.49	−2.67	nt	0.55	0.49	−0.74	−1.32	−0.50	−0.96
20	4.90	4.50	nt	nt	nt	nt	nt	nt	nt	nt	nt	nt	nt	nt	nt	nt	nt	nt
21	2.60	2.77	2.71	2.73	2.96	3.09	0.92	0.84	0.88	1.56	1.04	1.34	0.79	1.19	1.03	1.10	−0.35	0.35
23	3.14	3.04	3.16	3.71	3.58	3.80	0.85	0.85	0.83	−1.88	−2.07	−1.82	−1.86	−2.09	−1.70	−0.09	−0.03	−0.30
24	3.03	2.60	nt	4.12	3.19	nt	0.74	0.82	nt	−0.71	−1.80	Nt	0.76	−1.25	Nt	−2.04	−1.05	nt
25	2.75	2.65	2.67	3.19	3.18	3.12	0.86	0.83	0.86	−1.53	−1.76	−1.70	−1.64	−1.66	−1.78	0.21	−0.24	0.13
26	3.58	3.65	nt	4.02	4.00	4.09	0.89	0.91	0.89	0.27	0.43	0.45	0.17	4.00	0.28	0.01	0.42	0.13
29	2.79	2.90	2.88	3.40	3.61	3.65	0.82	0.80	0.79	−1.57	−1.63	−1.99	−1.24	−1.15	−1.43	−0.78	−1.01	−1.19
32	3.51	3.57	3.64	5.10	5.09	5.36	0.65	0.67	0.68	−2.42	−2.49	−2.04	−0.60	−0.88	−0.41	−2.66	−2.48	−2.36
33	2.02	1.83	nt	nt	nt	nt	nt	nt	nt	0.35	−0.46	nt	0.69	0.68	nt	−0.68	−1.72	nt
34	3.36	3.11	nt	3.93	4.00	nt	0.86	0.78	nt	0.43	0.91	nt	1.17	1.43	nt	−1.10	−0.79	nt
37	1.66	1.77	nt	4.45	4.42	nt	0.78	0.83	nt	nt	nt	nt	nt	nt	nt	nt	nt	nt
38	3.03	2.6	nt	2.32	2.37	nt	0.73	0.83	nt	nt	nt	nt	nt	nt	nt	nt	nt	nt

FEV_1_, forced expiratory volume in 1 sec; FVC, forced vital capacity; nt: not tested.

### Treatment outcome according to CD‐sens

**Figure 1:**
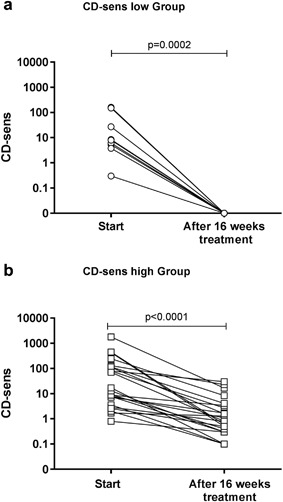
The individual CD‐sens values for all patients in the (a). CD‐sens low Group, *n* = 9 and (b). CD‐sens high Group, *n* = 23. All patients had a reduced CD‐sens and 9/32 (28 %) became CD‐sens negative. A statistical significance was considered at a *p*‐value of <0.05.

**Figure 2:**
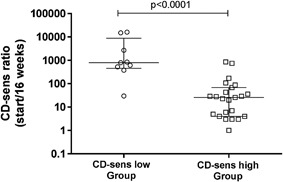
Difference in % CD‐sens decrease (ratio CD‐sens before treatment/CD‐sens after treatment) between CD‐sens low and CD‐sens high Groups. Results are expressed as median and inter quartile range and a statistical significance was considered at a *p*‐value of <0.05.

Nine of 32 patients (28%) became CD‐sens negative (<0.1) after 16 weeks of omalizumab treatment and were regarded as successful (CD‐sens low Group), while 23/32 did not become CD‐sens negative and treatment was regarded as unsuccessful (CD‐sens high Group) (Fig. 1 a and b) (Table 2 ). There was a significant difference (*p* < 0.0001) between the CD‐sens low and CD‐sens high Group in the percentage of CD‐sens decrease when comparing the CD‐sens before and after treatment (ratio CD‐sens before treatment/CD‐sens after treatment) (Fig. 2 ). The median ratios were 800 in the CD‐sens low Group versus 26 in the CD‐sens high Group.

### IgE sensitization

**Figure 3:**
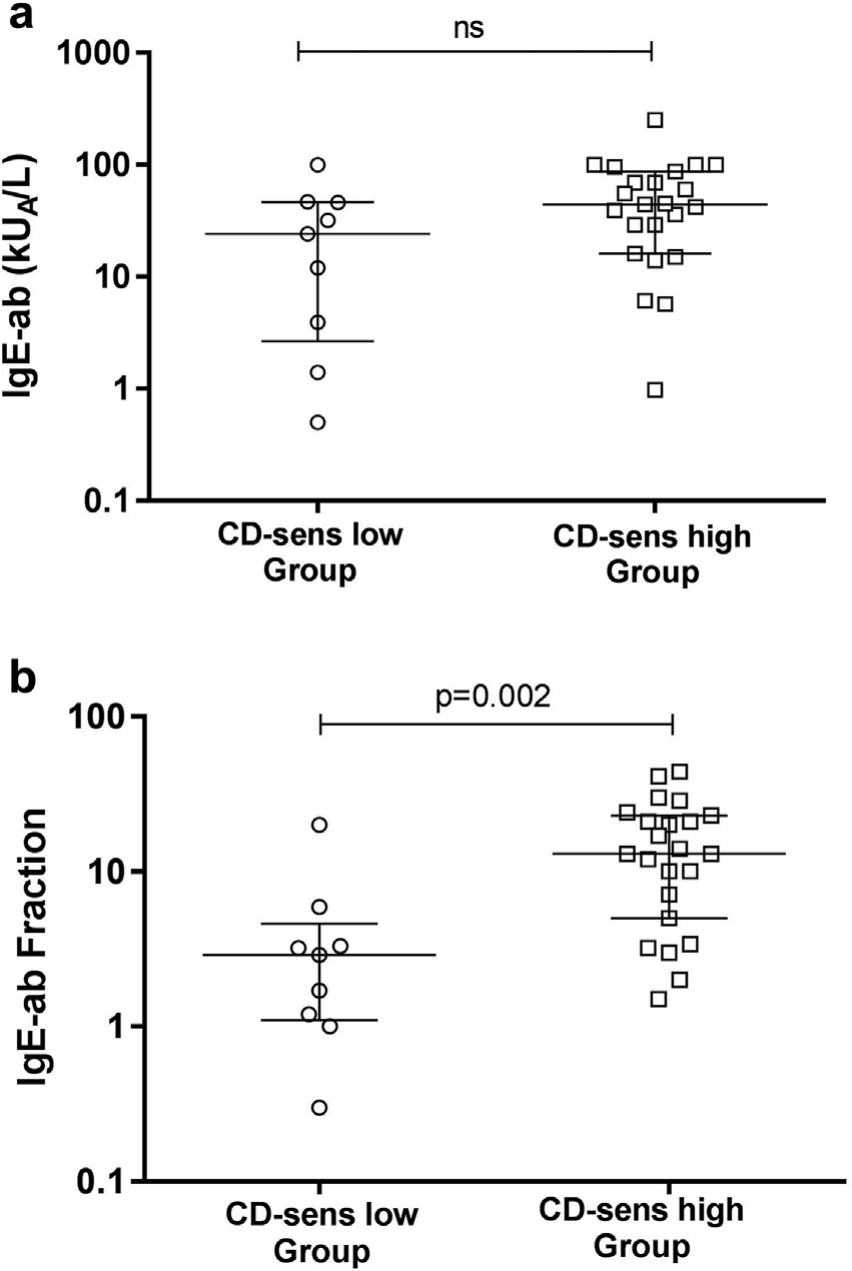
IgE‐ab to the most important allergen triggering the asthma was analyzed. (a) There was no significant difference in IgE‐ab values between the CD‐sens low and CD‐sens high Group. (b) There was a significantly smaller IgE‐ab fraction (IgE‐ab/IgE) in the CD‐sens low vs the CD‐sens high Group (*p* = 0.002). Results are expressed as median and inter quartile range and a statistical significance was considered at a *p*‐value of <0.05.

At inclusion, IgE‐ab were analyzed to the one inhalant allergen most important for triggering the asthma. There was no significant difference (*p* = 0.08) in IgE‐ab values (kU_A_/l) between the CD‐sens low and CD‐sens high Group. However, there was a significantly lower IgE‐ab fraction (IgE‐ab/IgE) in the CD‐sens low vs. the CD‐sens high Group (*p* = 0.002) (Fig. 3 a and b). In the CD‐sens low Group 7/9 tested patients (78%) had an IgE‐ab fraction <5% at the start of omalizumab treatment while in the CD‐sens high Group 5 of 23 tested patients (22%) had an IgE‐ab fraction <5% (Table 1 ).

### Skin Prick Test

SPT was performed before and after 16 weeks of omalizumab treatment in 24/32 patients (Table 2 ). The median ratios of the wheal area in mm^2^ before and after treatment (ratio SPT before treatment/SPT after treatment) with omalizumab was 1.9 in the CD‐sens low Group versus 1.4 in the CD‐sens high Group. There was no statistical significant difference (*p* = 0.82) between the two groups.

### FeNO

There was no statistically significant difference in FeNO before treatment between the CD‐sens low and CD‐sens high Group (*p* = 0.92). No change in FeNO levels between start and follow‐up was seen, neither in the CD‐sens low Group nor in the CD‐sens high Group (*p* = 0.43 and 0.06, respectively) (Table 3 ). In addition, there was no significant difference in the ratio of FeNO before and after 16 weeks treatment (ratio FeNO before treatment/FeNO after treatment) between CD‐sens low Group (median ratio 1.6) and CD‐sens high Group (median ratio 1.7) (*p* = 0.77).

### Spirometry

FEV1, FVC, and FEV1/FVC values were similar between the CD‐sens low and CD‐sens high Group at baseline (*p* = 0.45, 0.48, and 0.56, respectively) and no significant difference could be detected after 16 weeks of treatment in any of the two groups for FEV1 (*p* = 0.71 and *p* = 0.87, respectively), FVC (*p* = 0.32 and *p* = 0.53, respectively), or FEV1/FVC (*p* = 1.00 and *p* = 0.99, respectively) (Table 4 ).

Lung function was in addition calculated as z‐score FEV_1_/FVC (Table 4 ). No statistical significant changes in z‐score FEV_1_/FVC after 16 weeks of omalizumab treatment were observed neither in the CD‐sens low Group (*n* = 7) nor in the CD‐sens high Group (*n* = 17) (*p* = 0.90 and *p* = 0.84, respectively). Also, neither was there any significant difference in decrease in z‐score FEV_1_/FVC after 16 weeks of treatment (ratio z‐score FEV_1_/FVC before treatment/z‐score FEV_1_/FVC after treatment) between the two groups (*p* = 0.71).

### ACQ

ACQ was significantly higher before treatment in the CD‐sens low Group versus the CD‐sens high Group (*p* = 0.01) (Table 3 ). Further, there was a statistically significant decrease in ACQ after 16 weeks treatment with omalizumab in both the CD‐sens low Group and the CD‐sens high Group (*p* = 0.05 and 0.01, respectively). However, there was no significant difference in the ACQ ratio before and after treatment (ratio ACQ 1/ACQ 2) between the two groups (*p* = 0.23).

### GETE

There was no statistically significant difference in GETE between the CD‐sens low and the CD‐sens high Group after 16 weeks of treatment (*p* = 0.17) (Table 3 ).

### Increased omalizumab dosage

**Figure 4:**
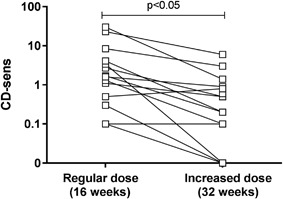
The individual CD‐sens values for all patients in the CD‐sens high Group after treatment with regular (16 weeks) as well as increased omalizumab dose (32 weeks). All patients had a reduced CD‐sens and 3/15 became CD‐sens negative. A statistical significance was considered at a *p*‐value of <0.05.

For 23 patients the treatment were regarded unsuccessful as judge by CD‐sens. For fifteen of these 23 patients the omalizumab dose was increased by 25–50% during an additional 16 weeks treatment that is, a total of 32 weeks treatment. CD‐sens decreased significantly (*p* < 0.05) and further 3/15 patients became CD‐sens negative (Fig. 4 ). After increased dose of omalizumab the median CD‐sens ratio (CD‐sens before treatment/CD‐sens after increased dose) increased from 26 to 81.

No further improvement could be detected for the tested clinical parameters SPT (Table 2 ), FeNO, ACQ, GETE (Table 3 ), or FEV1 and FEV_1_/FVC after increased omalizumab dose (Table 4 ).

## Discussion

Allergic asthma is caused by an allergen‐IgE‐ab mediated inflammation inducing a bronchial airway response. The presence of IgE‐ab in blood can be detected by in‐vitro immunoassay and their presence on the inflammatory cells that is, mast cells in the tissue and basophilic granulocytes in blood, by allergen provocation like skin tests and allergen inhalation.

Omalizumab has been available during several years for treatment of allergic asthma. Its mechanism is to bind free circulating IgE. Since there is a balance between free and cell bound IgE, the amount of IgE including IgE‐ab present on mast cells and basophils is reduced upon omalizumab treatment. As a consequence the initiation of the IgE‐ab mediated inflammation is blocked.

The clinically relevant allergen sensitivity in allergic asthma can be determined by bronchial allergen challenge, a rather complicated, and not risk free diagnostic procedure. By an in‐vitro dose‐response allergen stimulation of the basophils, their allergen threshold sensitivity, CD‐sens 16 can be accurately determined. Studies have shown that CD‐sens correlates significantly with bronchial allergen threshold sensitivity 9 , nasal allergen challenge 14, 18 , and oral food challenge 15, 19, 20 .

In the present clinical “real‐life” study, the efficacy of omalizumab has been evaluated by CD‐sens as an objective marker of the IgE‐mediated inflammation in parallel with clinical parameters like SPT, FEV1, FEV1/FVC, FeNO, ACQ, and GETE.

The response to omalizumab treatment was defined by CD‐sens. We found that 28% of the patients became CD‐sens negative after 16 weeks omalizumab treatment and they were regarded as successful (CD‐sens low Group). In addition, the CD‐sens decrease after treatment (ratio CD‐sens before treatment/CD‐sens after treatment) was much greater in the CD‐sens low Group, ratio 800, as compared to the CD‐sens high Group, ratio 26. By prolonging the treatment period in combination with an increased dosage another 20% of the CD‐sens high Group showed a positive efficacy of omalizumab treatment. However, despite a marked decrease in IgE‐mediated inflammation, as measured by CD‐sens, no conclusive improvement could be detected using clinical parameters like SPT, FeNO, FEV1, FEV1/FVC, ACQ, or GETE after 16 weeks or prolonged/increased dosage treatment. The likely explanation is that it takes a longer time for a bronchial inflammation to be eliminated. Most long‐term studies have focused on the safety aspect. A systemic review and a meta‐analysis from 2015 described the safety profile of omalizumab was excellent, the treatment was well tolerated, as only infrequent, and generally mild local reactions were observed following treatment. There were no drug‐related serious adverse events reported 21 . However, in a recently published study the additional improvement of the asthma after longer omalizumab treatment, that is, more than 16 weeks, showed that although the majority of patients responded already at 16 weeks (defined as “fast responders”) some patients did require longer treatment that is, 24 weeks (defined as “slow responders”) for more successful outcome 22 . Another study of omalizumab treatment efficacy on quality of life and FEV1% found that the efficacy gradually increased from baseline to 32 weeks and 4 and 9 years 23 .

It has previously been reported that in patients with a serum IgE concentration below 75 kU/L, no difference in asthma efficacy between treatment with omalizumab or placebo could be detected 3 . Patients with a low serum IgE concentration often have a large IgE‐ab fraction, that is, a high percentage of IgE‐ab out of IgE 24 . This could be the explanation for lack of efficacy of omalizumab, as it is likely that significant levels of IgE‐ab remain even if IgE is pressed down to 10 kU/L 25 . Using CD‐sens, a study of omalizumab in patients with a small or a large IgE‐ab fraction showed that the recommended omalizumab dose was not efficient if the IgE‐ab fraction was larger than 4% 26 . This was also confirmed in a study with intra nasal cat allergen challenge 27 . In the present study, we found a significantly smaller IgE‐ab fraction in the CD‐sens low Group, where 78% had an IgE‐ab fraction <5% at the start of omalizumab treatment, compared to only 22% for the CD‐sens high Group. Therefore, the size of the IgE‐ab fraction should be considered before start of omalizumab treatment, as it could indicate whether treatment with a regular omalizumab dose will be efficient or not.

We have previously shown, in patients with allergic asthma 24 or IgE‐mediated allergy to milk 28 or peanut 29 , that an increased omalizumab dose could further eliminate additional IgE‐ab and improve clinical symptoms not only from the lung but also from the nose, gastro‐intestinal tract, and dermis. We hypothesized that the recommended dose of omalizumab was too low in the 23 patients in the CD‐sens high Group in the present study. In 15 of these patients the omalizumab dose was increased by 25–50%. CD‐sens decreased significantly and further 3/15 patients became CD‐sens negative. In addition, the median CD‐sens ratio (CD‐sens before treatment/CD‐sens after increased dose) increased from 26 to 81 indicating a more successful inhibition of basophil activation and the IgE‐mediated inflammation. Even though the basophil allergen sensitivity decreased, as many as 80% (12/15) of the patients were still positive in CD‐sens, perhaps due to a low and moderate increase in omalizumab dose. We have previously reported an increase of the initial dose up to 400% to be necessary to eliminate clinical symptoms in severely peanut allergic patients 29 .

The results from this study show 72% of patients had an unsuccessful effect of omalizumab. This number was decreased to 62% after increased omalizumab dose. These numbers are higher than in a previous report from Bousquet et al. where 39% of the patients were reported as Non‐Responders as judged by clinical parameters 3 . The most likely explanation is that our small “real life” study consists of a heterogeneous group of patients with a severe and difficult to treat allergic asthma and a large IgE‐ab fraction. Although not as scientifically rigorous as clinical trials, these types of trials remain important in understanding how a therapy performs in the clinic 21 . The heterogeneity between studies may be a consequence of variations between different countries, different health care givers, from one clinical setting to another, and between different health care financing systems 30 . For financial reasons and practical purposes, omalizumab is usually not prescribed until conventional drugs have failed. Further, in severe allergic asthma, the non‐specific bronchial inflammation induced by IgE‐ab is severe. The IgE‐mediated part can be affected and controlled by omalizumab and evaluated by CD‐sens. However, even if the IgE‐mediated inflammation is eliminated, asthma symptoms dependent on the local, non‐specific bronchial reactivity will remain and should be treated accordingly.

In summary, despite a marked decrease in IgE‐mediated inflammation, as measured by CD‐sens, no conclusive improvement could be detected using clinical parameters like SPT, FeNO, FEV1, FEV1/FVC, ACQ, or GETE. This highlights the difficulties in evaluating omalizumab treatment effect and the importance of combining different diagnostic methods. By the use of the objective laboratory method CD‐sens, which effectively measures the direct effect of omalizumab, that is, the IgE‐mediated part of the allergic asthma, in combination with clinical parameters, it might be possible to more effectively monitor and treat IgE‐mediated allergic asthma.

## Authors' Contributions

All authors SGOJ, GJ, JH, and AN, conceived and designed the study; acquired, analyzed, and interpreted the data; drafted the article; wrote and critically revised the manuscript; and involved in the final approval of the version to be published.

## Conflict of Interest

SGOJ has received reimbursement for attending a symposia and research funds from Novartis Sweden. AN has received speakers fee and research funds from Novartis Sweden.
